# Elastometry of Complex Fluid Pendant Capsules

**DOI:** 10.1021/acs.langmuir.3c01845

**Published:** 2023-11-08

**Authors:** Amy Z. Stetten, Felix S. Kratz, Nathalie Schilderink, Subhash Ayirala, Michael H. G. Duits, Jan Kierfeld, Frieder Mugele

**Affiliations:** † Physics of Complex Fluids Group, University of Twente, 7500 AE Enschede, The Netherlands; ‡ Department of Physics, TU Dortmund University, 44221 Dortmund, Germany; § EXPEC Advanced Research Center, Saudi Aramco, 34465 Dhahran, Saudi Arabia

## Abstract

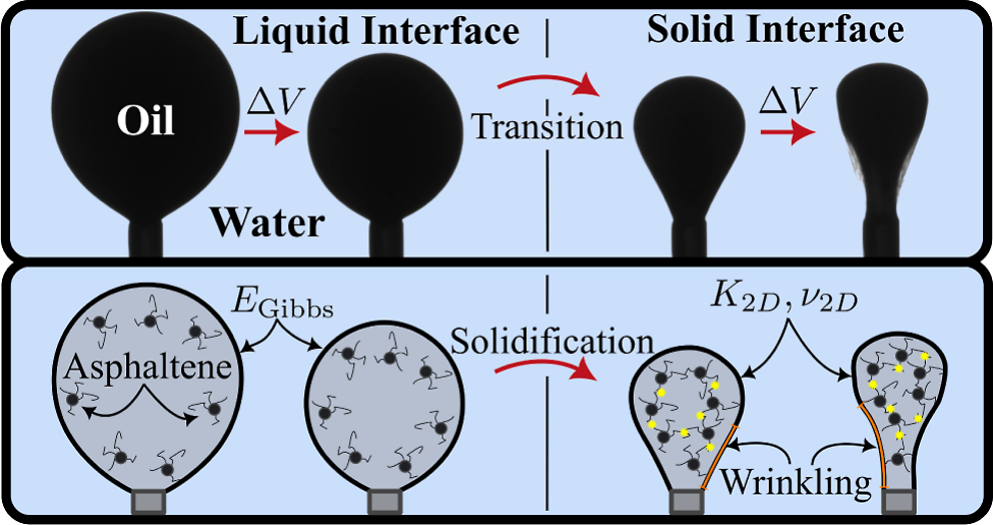

Oil/water interfaces are ubiquitous in nature. Opposing polarities at these interfaces attract surface-active molecules, which can seed complex viscoelastic or even solid interfacial structure. Biorelevant proteins such as hydrophobin, polymers such as PNIPAM, and the asphaltenes in crude oil (CRO) are examples of some systems where such layers can occur. When a pendant drop of CRO is aged in brine, it can form an interfacial elastic membrane of asphaltenes so stiff that it wrinkles and crumples upon retraction. Most of the work studying CRO/brine interfaces focuses on the viscoelastic liquid regime, leaving a wide range of fully solidified, elastic interfaces largely unexplored. In this work, we quantitatively measure elasticity in all phases of drop retraction. In early retraction, the interface shows a fluid viscoelasticity measurable using a Gibbs isotherm or dilatational rheology. Further retraction causes a phase transition to a 2D elastic solid with nonisotropic, nonhomogeneous surface stresses. In this regime, we use new techniques in the elastic membrane theory to fit for the elasticities of these solid capsules. These elastic measurements can help us develop a deeper understanding not only of CRO interfaces but also of the myriad fluid systems with solid interfacial layers.

## Introduction

Oil/water interfaces are ubiquitous in nature. Because many organic molecules are surface active, they have the potential to migrate, accumulate, and seed complex structures at such interfaces. This can lead to the formation of viscoelastic liquid interfaces or even solid interfacial layers. Biorelevant proteins such as hydrophobins,^1^ industrially intriguing polymers such as PNIPAM,^2^ and energy-relevant crude oils (CROs)^3^ are some examples of systems where such layers occur. Solidifying the interface between two liquids results in dramatic changes in the system behavior. One can imagine changes in capillary pressures, fluid flows, emulsion stability, adhesion properties of the interface, transport between the phases, and many other fundamental properties.^4^ In order to understand and control these systems, we must be able to make quantitative measurements of the rheology of truly solid elastic interfacial layers. In this work, we focus on the interface between CRO and brines containing aqueous salt ions. Such interfaces occur naturally within oil reservoirs and unnaturally in ocean oil spills. CROs contain an incredible diversity of surface-active organic molecules that can result in a very interesting and complex interfacial rheology. This interfacial rheology of the brine/CRO interface can play a significant role when one attempts to recover the oil or separate the two phases.^5^ Oil recovery is well-studied, and it is well-known that switching from early stage flooding with high-salinity water to later-stage, lower-salinity water yields an incremental increase in oil recovery. However, the reasons for this improved recovery remain somewhat elusive.

The molecules believed to be responsible for the complexity of CRO/brine interfacial layers are the asphaltenes. Asphaltenes are a solubility class of amphiphilic, polyaromatic species defined as being soluble in toluene but insoluble in *n*-alkanes. The amphiphilic nature of the asphaltenes in combination with the significant alkane fraction in most CROs leads the asphaltenes to adsorb (often irreversibly) to the oil/brine interface.^6^ As asphaltenes adsorb, they form a viscoelastic fluid interfacial layer that may, under the right conditions, undergo a phase transition into a stiff solid interface. The packing and structure of these asphaltene layers depends on many factors including resin content, ionic composition of the brine, hydrogen bonding, temperature, surface history, kinetics, and other factors.^7^ A recent, thorough review by Moud discusses the importance of various controlling factors in asphaltene layer development at the oil/brine interface.^8^

There are two primary ways in which asphaltene-induced interfacial rheology could affect oil recovery. The first one is through the connectivity of the oil phase. Interfacial layers could act as a barrier to oil snap-off, thus keeping the oil phase more connected during reservoir flooding, leading to reduced pressure fluctuations and greater oil recovery.^9^ Bidhendi et al. show a series of compelling experiments suggesting that the role of rheology might even dominate over the more traditionally accepted wettability alteration.^10^ As is often the case in complex systems, there is conflicting evidence as to whether these stiffer films have a positive or negative effect on oil recovery. Some suggest that these films make it more difficult to destabilize oil/water emulsions, leading to higher pressure-heads and clogging of small reservoir pores.^11^

The second way in which interfacial rheology could affect oil recovery is through the deposition of the oil/brine interfacial layers onto the mineral surface. Many CRO components (asphaltenes, resins, complexes, etc.) are sparingly soluble in water; thus, wettability alteration of the rock surface could occur even through a thin water layer. The model developed by Kaminsky et al.^12^ finds that the equilibrium surface coverage achieved by diffusion through a water layer is not enough to reverse wettability. However, they suggest that natural flow could cause puncturing and deposition of the oil/brine interfacial film onto the mineral surface, altering the mineral wettability.^12^ If this is true, then the character and thickness of these oil/brine interfacial layers could lead to significant differences in the wettability of the mineral structure.

One of the most interesting things about CRO/brine interfaces is the long list of variables that control the surface rheology. In order to get an idea of these controlling variables, we will briefly outline the reported effects of aging time, brine composition, oil composition, and temperature on CRO/brine interfacial tension (IFT) and interfacial rheology (dilatational and shear rheologies generally yield comparable results).

### Aging Time

The literature is in agreement that viscoelasticity of CRO/brine interfacial layers increases with aging time.^3,11,13−18^ Also undisputed is the fact that, after some short initial aging time, the elastic modulus dominates the viscous modulus.^3,9,16,19^ Asphaltene adsorption is generally seen to be diffusion controlled at early times and to include more kinetic surface structure reorganization at later times.^20^

### Brine Composition

There is less literature addressing the role of brine composition in interfacial rheology, but the literature does show that the stiffest surfaces are seen at low salt concentration.^3,10,15^ Asphaltenes contain both positively and negatively charged groups, which tend to interact with groups of opposite charge. A strong ionic brine could shield these interactions, preventing or slowing the self-association of the asphaltenes and softening the resulting layer. The trend with ionic strength is not always seen to be monotonic, often there is a peak with increasing salt concentration in IFT,^21^ elasticity,^9,16^ or both. Additionally, some have seen specific ion interactions.

### Oil Composition

Higher asphaltene content in CRO yields a stiffer oil/brine interface.^17^ The addition of natural resins can complicate this trend since resins may solubilize asphaltenes, inhibiting skin formation at high resin concentrations.^20^ In addition to the competition with resins, the degree to which asphaltenes can lower the IFT of a CRO/brine interface is strongly dependent on solvent quality, suggesting that asphaltenes orient differently depending on the composition of a particular CRO.^13^

### Temperature

The effect of the temperature on interfacial asphaltene layers is ambiguous. There is evidence that the IFT of CRO/brine interfaces decreases^21,22^ or increases^15,19^ with the temperature. In terms of viscoelasticity, the same ambiguity arises. Some researchers show that higher temperatures yield faster layer growth.^16^ Others have seen that higher temperature shows lower interfacial viscosity.^23^ There are even those who see no change in viscoelasticity with temperature whatsoever.^14^ We do not claim to enter this debate with an answer; the answer is likely to be that the individual systems are different enough in composition and history that they may all be correct. We will, however, present one reason why this sort of ambiguity is quite likely to occur.

Asphaltene layers grown under a wide variety of the above-mentioned conditions show distinct phase transitions when compressed. Both Yarranton et al.^14^ and Kabbach and dos Santos^24^ nicely describe the surface pressure versus area “isotherms” for these interfaces and the distinct slope change that indicates a phase change of the interface during compression. With the assumption that asphaltenes adsorb nearly irreversibly to oil/brine interfaces, the slopes of such isotherms define Gibbs elasticity. This reveals a softer phase and a stiffer phase. Continuing to compress beyond this stiffer phase induces surface crumpling and distortion that they attribute to a solid interfacial layer.^3,14^

While everyone seems to agree that asphaltene layers can be solid, the microscopic picture of these solid layers is yet debated. Many assume the layer to be a cross-linked gel-like membrane; some say it is a glassy jammed solid. Measurements of these layers at 2–9 nm thick seems to suggest that network formation extends into the oil phase.^25^ Additionally, interfacial shear rheology and particle tracking have shown that asphaltenes form rigid, heterogeneous films that immobilize particles on the surface.^26^ Such heterogeneous films would be less likely for a jammed solid, which would be free to redistribute surface stresses until the moment of jamming.

On the other side, a number of authors have shown that asphaltene–laden interfaces show a unique equation of state (EoS), meaning every IFT corresponds to a unique surface coverage, even independent of external conditions. They also state that the maximum surface coverage aligns well with the average size of a single asphaltene molecule, suggesting a packed, but unaggregated, asphaltene layer.^27−30^ Others find a single EoS when the heptane fraction in the solvent is low, but suggest gel formation when the heptane fraction is higher (as it is in natural CRO) perhaps due to adsorption of nanoaggregates to the interface.^31^ Some authors also propose both an EOS model at lower asphaltene coverage and a solid model at higher coverage.^32^

In the references discussed so far, the researchers have explored CRO/brine interfacial rheology using different techniques for viscoelastic fluid interfaces such as Gibbs isotherms, dilatational rheology, shear rheology, and others. These methods tend to run into issues when used on surfaces that have formed truly solid layers.^4^ Once the layer has undergone a phase transition to fully solid, the interfacial stresses are no longer homogeneous and isotropic and Young–Laplace fits are no longer viable, so many of these techniques break down.^11^ Previous authors have used the onset of Young–Laplace fit errors to describe a potential phase transition; however, this method is qualitative.^33^ Recently, the detection of deviations from Laplacian shapes without (computationally intensive) fitting to them has also been described.^34^

In this paper, we introduce a new method for quantitative measurements of the elasticity of a fully solidified CRO/brine interface. We simulate the “pendant capsule” shapes of droplets with an elastic shell using the elastic membrane theory and then fit these shapes to our experimental data. These simulations suggest a solid phase transition that occurs far earlier than is discussed in most previous work. By comparing the residual error between fits utilizing the Young–Laplace equation and fits using the elastic shape equations, we are able to determine the importance of anisotropic and inhomogeneous surface stresses that arise upon sufficient deflation.

The interface between oil and water is one of the most thoroughly studied interfaces in the literature; however, any number of added organic molecules or colloidal particles could cause these pure liquid interfaces to grow elastic solid interfacial layers. Having access to the elastic properties of such interphases will give us deeper insights into and greater control over how such systems behave.

## Methods

### Experimental Methods

CRO samples were obtained from a carbonate reservoir. Characterization of CRO batches was performed by a certified laboratory at Saybolt Nederland B.V., and viscosity was measured with a Haake RS600 controlled stress rheometer using a Couette geometry (results as reported in ref (35) reproduced in Supporting Information, Table S1). CRO samples were stored in airtight containers and agitated before each use to promote homogeneous distribution of heavier components. CRO was used as-is and was not diluted or filtered for these experiments.

Deionized (DI) water came from a Millipore Synergy instrument with a water conductivity of 18.2 MΩ cm. Salts (NaCl, CaCl_2_, MgSO_4_, and NaHCO_3_) and organic cleaning solvents (ethanol, 2-propanol, acetone, and toluene) were purchased from Sigma-Aldrich and used as received. Artificial brines were prepared with the ion compositions listed in Supporting Information, Table S2. Salts were dissolved overnight under magnetic stirring at room temperature, followed by filtering through a 0.45 μm polyether-sulfone membrane. The four brines are formation water (∼4 M), high salinity injection water (∼1 M), 10× diluted high salinity injection water or “SmartWater” (∼0.1 M), and DI water. For simplicity, we will refer to the brines by their approximate molarity in parentheses above.

**Figure 1:**
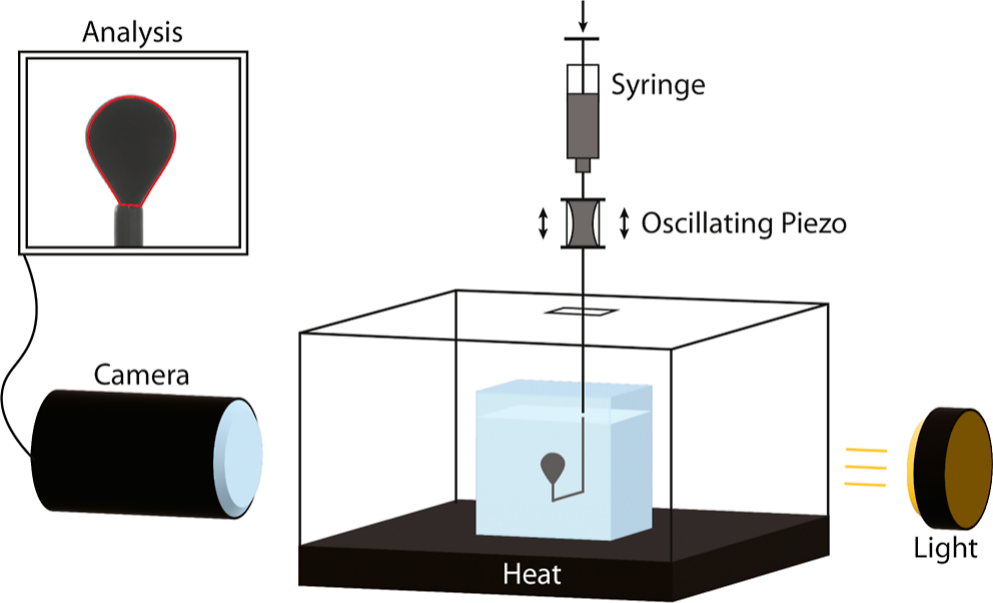
Schematic of the experimental setup. The sample was placed in a heat-resistant cuvette within a heating box. Heat is provided from below. A syringe mounted above is filled with CRO, which is pushed through a u-shaped syringe needle to form a pendant drop. A wide piece of tubing held by an oscillating piezo is used to sinusoidally change the volume of the drop during oscillating measurements. The sample is backlit and recorded by a camera linked with analysis software.

Pendant drop and oscillating pendant drop analyses were performed on a DataPhysics Optical Contact Angle Goniometer running SCA20 software. This setup consisted of a vertical syringe mount housing a 100 μL Hamilton syringe attached to a small piece of oscillation tubing held between two piezo-actuated mountings (Figure 1). The piezo could be activated using a piezo actuator (Piezosystem Jena ENT400/ENV800 controlled by DataPhysics ODG 20AMP) and could be fed any oscillating function using a function generator (Agilent 33220A 20 MHz Arbitrary Waveform Generator, California, USA). Below the oscillation tubing was a u-shaped syringe used to produce and hold the pendant drop. The tip of this u-shaped syringe was submerged in a glass cuvette of brine before the production of a pendant drop. If the piezo-actuator was turned on, the drop volume would oscillate sinusoidally. Large volume adjustments (producing a drop, retracting a drop, and inflating a drop to release it) could be controlled through DataPhysics software, which controlled the piston of the mounted syringe. Bottom heating was controlled through a circulating water bath heater (Haake Technik GmbH, Germany) and measured using a temperature sensor (Thorlabs) touching the side of the glass cuvette (not submerged in the liquid simply because cleaning CRO requires toluene, which would damage the sensor).

During these experiments, a horizontally mounted camera recorded the changing drop shape. With the input of liquid densities (Supporting Information, Table S3) and syringe diameter (0.5 or 0.45 mm), DataPhysics software allowed for Young–Laplace fitting of all liquid-like drop shapes and recorded IFT, fit error, drop volume, and apparent surface area for every frame.

The general experimental procedure was as follows. The Hamilton syringe was loaded with fresh CRO. The glass cuvette was filled with the chosen brine and placed on the heating stage with the lid closed. Heating was set to the desired temperature, and the system was left to equilibrate (until the temperature was stable for 10 min). The u-shaped syringe was lowered into the brine, and the DataPhysics controller was used to push air out of the syringe and to produce a CRO drop. Fresh CRO drops were used for every experiment so that we could monitor the effect of surface aging. At high temperatures, a thin layer of hexadecane was placed on top of the surface to minimize evaporation. The drop was left to age for the desired aging time. During this aging, the piezo was turned on for brief moments in order to oscillate the drop and record the viscoelastic moduli. After aging was complete, the drop was retracted at a constant rate of 0.1 μL/min until it completely crumpled. In some experiments, the drop deflation was paused at intermediate volumes to take oscillating measurements. At the end of the experiment, the drop was inflated until it broke off. The camera was recorded throughout the entire experimental procedure at a frame rate suitable for analysis of the results (lower frame rate during long aging periods, higher frame rate for oscillations).

### Liquid Droplets and Their Shapes

**Figure 2:**
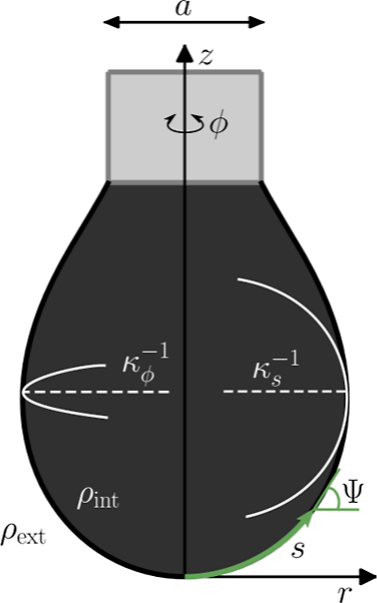
Visualization of the geometrical parameters used to formulate the shape equations.

As in previous studies,^1,36,37^ we will consider axisymmetric pendant liquid droplets that can be constructed by rotation of a single curve *C* around a symmetry axis, which we choose to be the *z*-axis. The curve *C* is parametrized by a function *r*(*s*), where *r* is the radial distance to the symmetry axis and *s*(*r*, *z*) the arc length such that d*s*^2^ = d*r*^2^ + d*z*^2^. The total arc length of *C* is given by *L* = ∫_0_^*L*^d*s*. Additionally, we define the arc angle Ψ as the angle between the tangent at the *r*(*s*) and the *r*-axis. For a visual presentation of these geometrical quantities, refer to Figure 2.

An axisymmetric liquid droplet shape is fully characterized by three *shape equations*. Two of those shape defining equations are of purely geometric origin
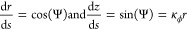
1where we have introduced the circumferential curvature κ_ϕ_ ≡ sin Ψ/*r*. The third and final shape defining equation can be found by energy minimization, or equivalently, local force balance.^37^ The result is the well-known Young–Laplace equation with a hydrostatic pressure load
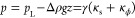
2where we introduced the height-dependent hydrostatic pressure with the pressure difference *p*_L_ across the interface at the apex of the droplet, the density contrast Δρ between inner and outer phase, and the gravitational constant *g*. It relates the hydrostatic pressure to surface tension γ and the mean curvature, where κ_*s*_ ≡ dΨ/d*s* is the meridional curvature. Rearranging 2 gives the final shape equation
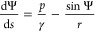
3

We choose to nondimensionalize the system with the diameter of the capillary *a* and the surface tension γ, which gives two independent, continuous, nondimensional control parameters
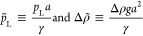
4and a third discrete control parameter Ω, which indicates how many necks and bulges a shape has (see ref (37) for details). Vice versa, surface tension and apex pressures can be obtained for given nondimensional control parameters  and  via
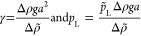
5where we need information about the diameter of the capillary *a* and density contrast Δρ to extract the relevant dimensional quantities.

Finally, we are able to solve 1 and 3 by integrating the equations from the apex to the capillary via a fourth order Runge–Kutta method. We utilize initial conditions and boundary conditions *r*(*s* = 0) = Ψ(*s* = 0) = *z*(*s* = 0) = 0 and *r*(*s* = *L*) = *a*/2. The numerical singularity in 3 for *s* → 0 can be cured by explicitly evaluating the limit dΨ/d*s*(*s* → 0) = *p*_L_/2γ.

### Fitting Liquid Drops

From the pendant drop experiment, we obtained a target shape *S*_T_. The objective is to find a set of parameters , which generates a shape  that minimizes a suitable error metric constructed from an error vector  comparing a number dim *E⃗* of shape coordinates from both shapes
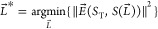
6

This generates a parameter set  that optimally approximates a solution of . In our fits to experimental shapes, we compare dim *E⃗* = 250 points distributed equidistantly in arc length and specify the residual fit error by the root-mean-square error  for the optimal parameter set (see Figure 6c below).

**Figure 3:**
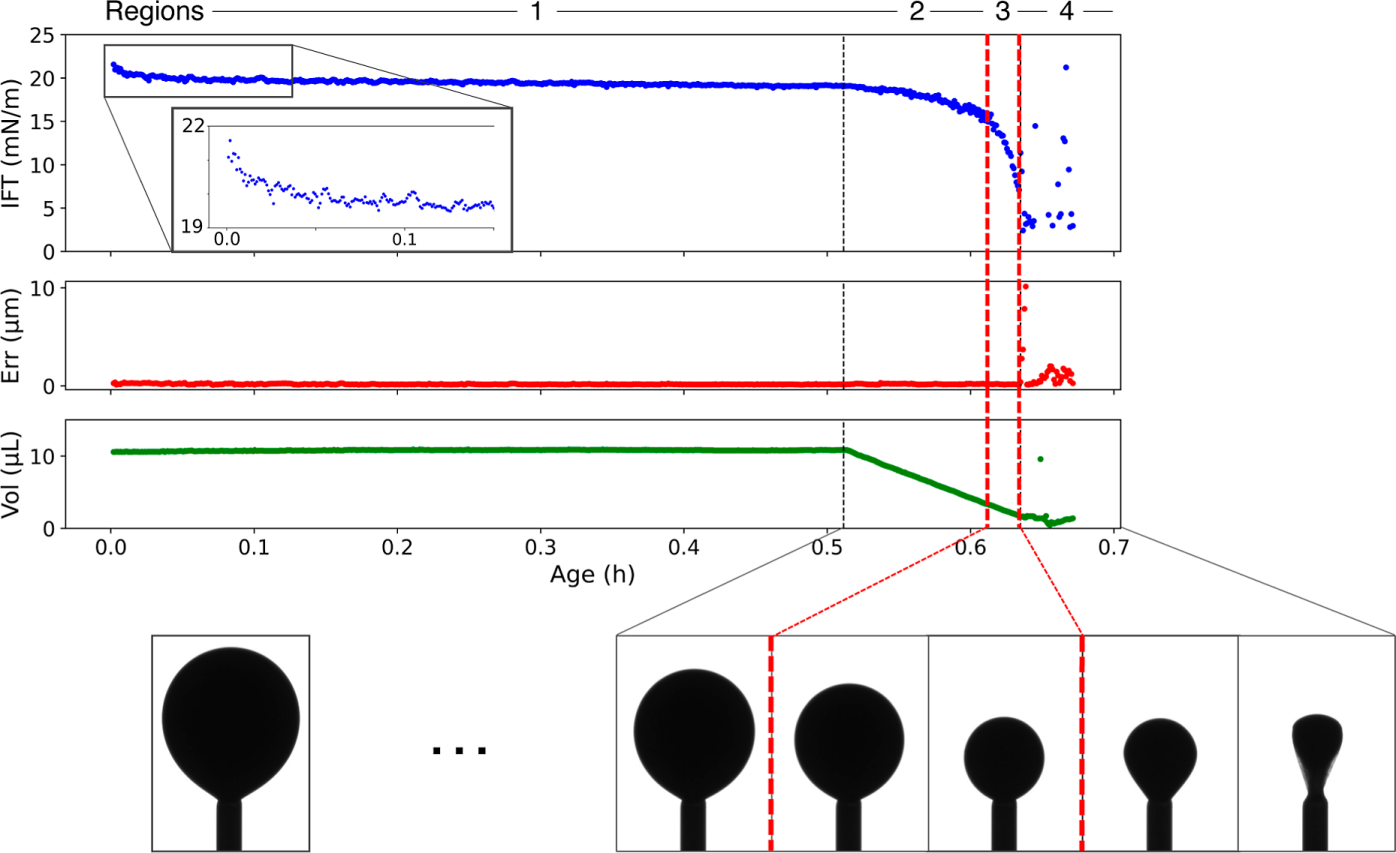
Results from a typical CRO pendant drop experiment in brine. This particular experiment shows CRO aged for 30 min in DI water at 60 °C. In region 1, the drop ages for 30 min at constant volume. It shows a slight initial decrease in IFT and then a plateau. In region 2, the drop is retracted at a constant rate. Continued constant retraction into region 3 shows a change in slope of the surface pressure isotherm. In the final “crumple” region, the drop interface visually deforms, wrinkles, and crumples entirely.

**Figure 4:**
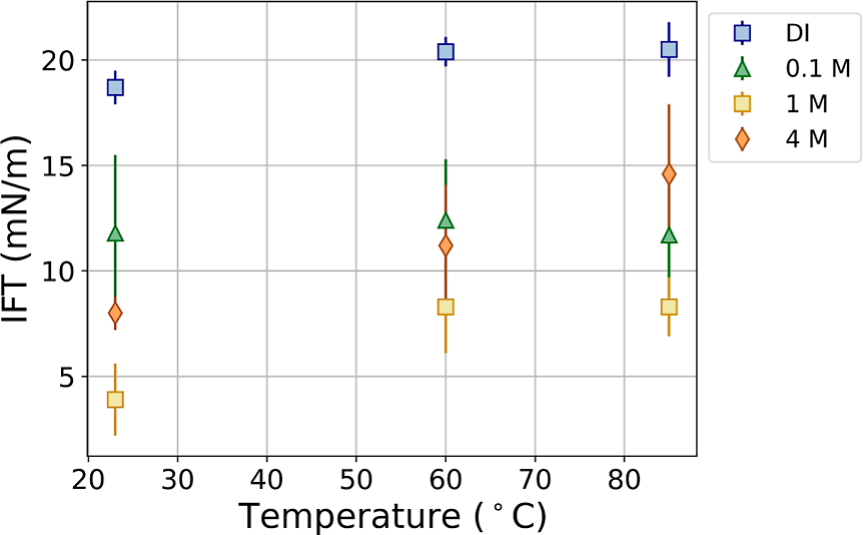
IFTs measured as the average between 25 and 30 min of aging. Two trends are evident. First, the IFT almost always increases with temperature. Second, the IFT decreases with increasing brine concentration with a minimum at 1 M (high salinity injection water).

**Figure 5:**
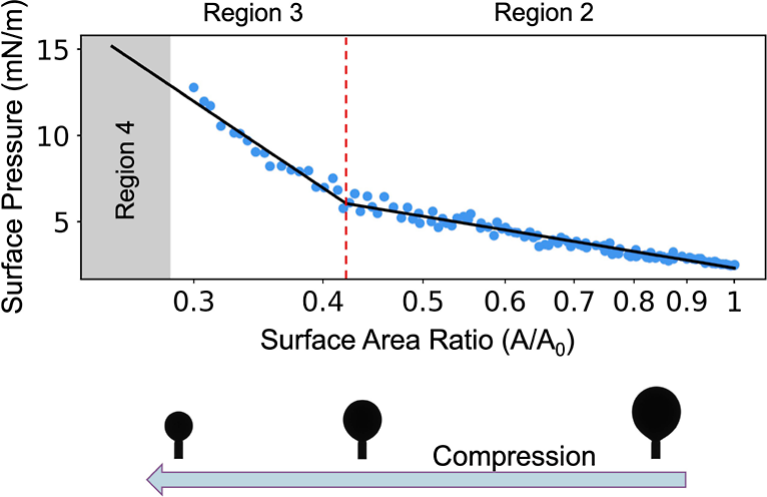
Example surface pressure vs surface area ratio isotherm for a CRO/DI water interface aged for 4 h at 60 °C. Note that the *x*-axis is plotted logarithmically, so the slope in this plot is the Gibbs elasticity. As the drop is retracted from right to left, the relative surface area decreases, and the surface tension goes down (surface pressure goes up). We see a distinct change in the Gibbs elasticity during retraction, suggesting a phase transition on the surface.

**Figure 6:**
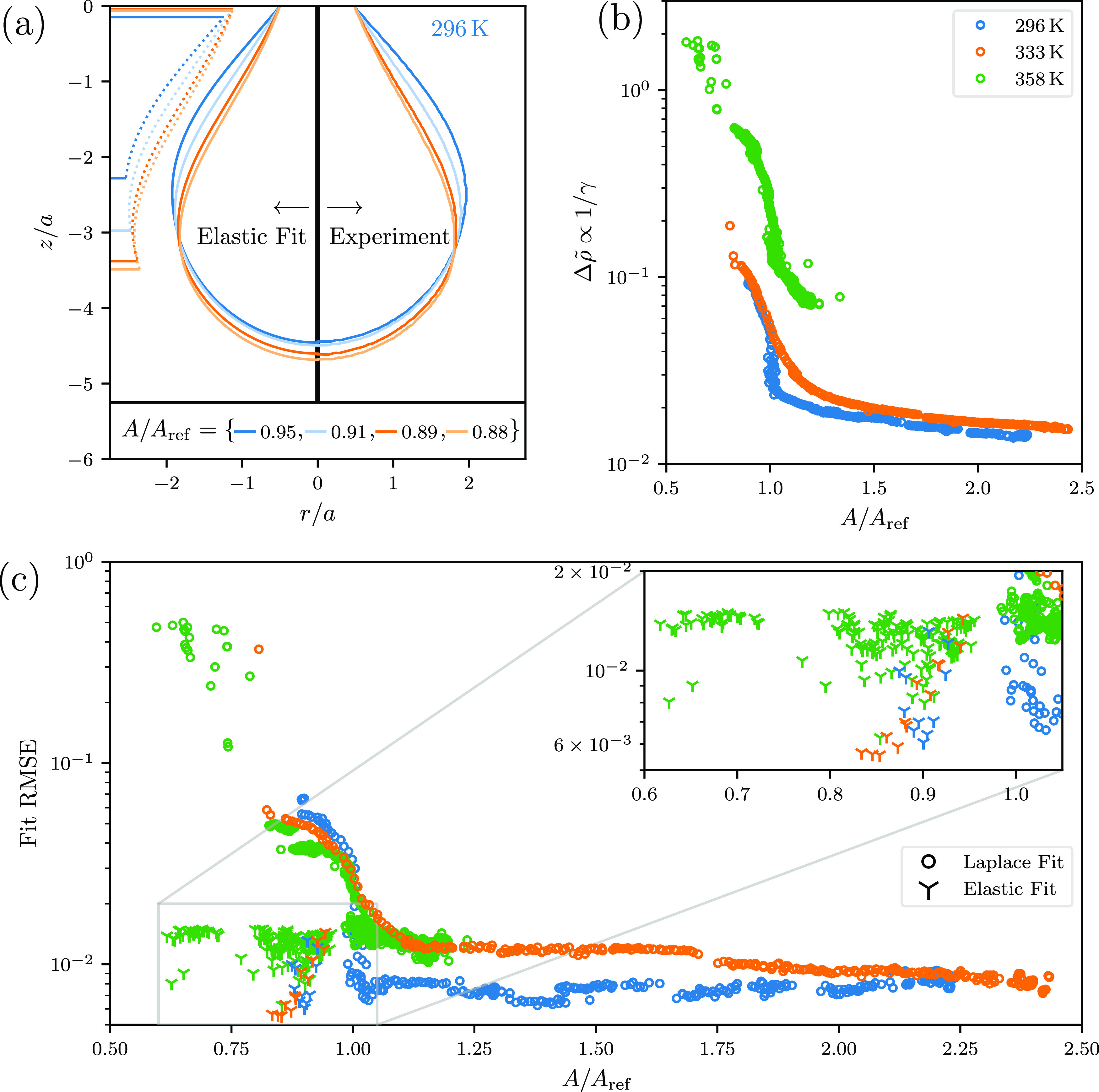
We perform two kinds of fits: a purely liquid (Laplace) fit and an elastic fit. We show the relevant control parameters as a function of the relative area compression *A*/*A*_ref_, where *A*_ref_ is the area of the shape after which the shape error (RMSE) for Young–Laplace fits starts to drastically increase, as can be seen in (c). The secondary increase in fit error as seen prominently for the green points at strong area compression *A*/*A*_ref_ < 0.8 is of technical nature, since the solution class changes from Ω = 2 to Ω = 3 and our Young–Laplace fit only considers shape class Ω = 2 solutions. The nondimensional control parameter Δρ̃ is shown in (b), it is the more important one, since we use it to acquire the dimensional surface tension. Figure (a) shows a visual comparison of the shapes seen in experiments (right) and the best fitting theory shapes (left). Additionally, the dotted lines on the left of (a) indicate the effective shape of the wrinkled region. The shape error (RMSE) of the elastic fit is detailed in the inset of (c) and is always lower than the error achieved by a Young–Laplace fit. Finally, the elastic compression modulus, determined from the fits of the experimental images, is shown in Supporting Information, Figure S3c. Importantly, each point in any of the figures is generated by performing *two* independent fits for both hemispheres of the experimental image and averaging those results weighted by their respective fit error. The additional control parameters acquired from the fit, i.e., the dimensionless apex tension, dimensionless reference apex pressure, Poisson’s ratio, and compression modulus, are shown in Supporting Information, Figure S3.

We solve this kind of least-squares problem by a Newton-like algorithm, which iteratively searches for the zero of the error vector  and utilizes information about the derivatives in the error-shape landscape (i.e., the Jacobian matrix ) at the current guess  to generate an improved guess .^1^ We calculate the Jacobian numerically by solving the shape equations twice in the direct vicinity of the current parameter set . We update the parameter set in each iteration of the algorithm and employ a line-search algorithm to enforce  by eventually backtracking through  with ξ ∈ (0, 1) until the condition is fulfilled.

An alternative to this iterative numerical procedure would be to find an approximate inverse mapping L* = *S*^–1^(*S*_T_) by machine learning methods, eliminating the need for computationally expensive iterative techniques at the cost of traceability, as shown in ref (37) where a mapping for *S*_0_^–1^ is found using a feed-forward neural network.

### Elastic Capsules and Their Shapes

For an elastic capsule, that is, a droplet with a two-dimensional elastic skin, the surface stresses become anisotropic and inhomogeneous,^36,38^ and we obtain additional equations that govern the shape. We still assume axisymmetric shapes but can soften this condition to also allow circumferentially wrinkled shapes.^36^

For a purely elastic capsule we define the surface energy density *w*_*S*0_(λ_*s*_,λ_φ_) measured per *undeformed* unit area as a function exclusively of the meridional and circumferential stretches λ_*s*_ and λ_ϕ_. The appropriate choice for the meridional and circumferential surface stresses τ_*s*,ϕ_ is thus^36^
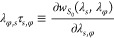
7

The shape equations again minimize the appropriate energy functional
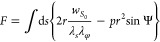
8

The first variation of the energy functional leads to the nongeometric shape equations (for details, see refs (1) and (36))
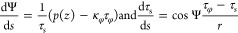
9

Since we are now considering an elastic interface material, we need to specify a constitutive model and a reference shape *S*_0_. The constitutive model is chosen to be accurate up to quadratic order with respect to the surface energy density, while still incorporating the geometric nonlinearities of the problem^1,36^
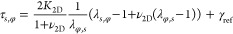
10where we implicitly choose the reference shape to be a liquid drop with tension γ_ref_ and introduce the circumferential/meridional stretching ratios λ_ϕ_ and λ_*s*_, the two-dimensional compression modulus *K*_2D_ = *Y*_2D_/[2(1 – ν_2D_)] (*Y*_2D_ is the two-dimensional Young’s modulus) and the two-dimensional Poisson number ν_2D_. A special treatment is necessary for compressive circumferential stresses τ_ϕ_ < 0. Compressive stresses *must* be relieved via wrinkles at the surface of the capsule if the thickness of the interface is small, since solutions including regions of compressive stress are unstable.^39^ The resulting wrinkles relax the tension and lead to an effective shape where the circumferential stress is pinned to zero in the region, where it would be negative in the unwrinkled shape:^1,36^.

Including the geometric nonlinearities in the surface stresses 10 and allowing circumferential wrinkles is an improvement made in ref (36) over the theory presented in ref (38). Moreover, we also employ a systematic fitting of experimental shapes by the theoretically predicted shapes as it was introduced in refs (1) and (36).

The nondimensionalization follows the exact same scheme as for the liquid drop; thus, we arrive at the nondimensional control parameters for the shape of an elastic pendant capsule with a liquid reference: . The apex stress τ_*s*_(*s* = 0)/γ_ref_ appears as a control parameter since we have no initial condition for it, and it controls the apex pressure of the deformed shape. Using the apex pressure of the deformed shape as a control parameter instead of the apex stress might seem equivalent, but the mapping between apex stress and apex pressure is not bijective and can, thus, not be inverted without taking care of the ambiguity that arises necessarily: any choice of τ_*s*_(*s* = 0)/γ_ref_ generates a unique shape with an appropriate apex pressure, while any choice of apex pressure does not generate unique shapes with some appropriate apex stress, there are multiple valid shapes for some choices of an apex pressure with different apex stresses, as can be seen even in the case of Δρ̃ = 0.^40^ This is a conceptual problem encountered in prior publications that resulted in an inaccessibility of solution branches, making a fit to experimental data inherently unstable and ill-conditioned.

We will integrate the shape equations with respect to the reference, *undeformed*, arc-length *s*_0_, which requires us to perform a change in variables d/d*s* → λ_*s*_^–1^d/d*s*_0_. Now it is possible to integrate the shape equations along with the constitutive eq 10 by utilizing a shooting method to search for a valid apex pressure, connecting the deformed arc with the capillary and thus providing a solution for the attachment boundary condition *r*(*s* = *L*) = *a*/2. The boundary and initial conditions are exactly the same as for the liquid drop except that we have τ_*s*_(*s* = 0)/γ_ref_ as an additional control parameter in this case.

### Fitting Elastic Capsules

The fit for an elastic capsule is separated into two parts, first, we fit the pendant liquid drop reference shape to get access to the parameters .

Second, we utilize the same machinery as previously discussed for eq. 6, except that we now have a slightly modified problem statement: let  be a parameter vector for the elastic problem modulo the reference degrees of freedom (i.e., ) and *S*_L_ be a mapping from the elastic parameter space to the elastic shape space with given reference parameters L⃗. The parameter set  is considered to be the best elastic fit for some target shape *S*^T^ if it is a solution to
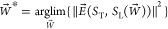
11

Since 6 and 11 are from the same class of problems, we solve it by utilizing the exact same procedure as discussed for the liquid drop. In our fits to experimental shapes, we specify the residual fit error by the root-mean-square error  for the optimal parameter set (see Figure 6c below).

We use a significantly improved version of the solver published in ref (1) to fit the experimental shapes. One major improvement is using the apex pressure as the shooting parameter instead of the apex stress, which improves the stability of the shape fit tremendously. A description of all improvements to the code and the new code will be released separately at a later point.

## Results and Discussion

A typical CRO pendant drop-capsule experiment is shown in Figure 3. It can be divided into four distinct regions: (1) aging of the liquid interface at constant volume, (2) early retraction with a lower Gibbs elasticity, (3) later retraction with a higher Gibbs elasticity, and (4) and eventual collapse and crumpling. The four regions of this single experiment provide a wealth of information about the different surface phases available under the given aging, temperature, and brine conditions of the experiment. We will first discuss each region of a typical experiment and the corresponding analysis used. We will then apply this type of experiment to a broad parameter space of ambient conditions.

### Region 1—Aging at Constant Volume

During aging, the drop interface remains fluid (meaning that the shape can be fit by the Young–Laplace equation with an error below 10 μm). The Young–Laplace fits yield an evolving IFT that decreases slightly over the first approximately 15 min, likely due to the adsorption of surface-active moieties to the interface. This is in agreement with the literature. In most cases, the IFT saturates and remains roughly constant (on the time scale of our experiments). Averaging over this saturated region for drops aged in various conditions reveals IFT trends with temperature and brine concentration (shown in Figure 4). With an increasing temperature, the IFT increases. This is somewhat uncommon, but not without example in the literature.^15,19^ A possible explanation could be that some CRO molecules desorb from the liquid–liquid interface, in relation to their lower affinity with the interface.^41,42^ Our experiments showed consistently increasing IFT whether the temperature was increased between experiments or over the course of a single experiment. Increasing the brine concentration reveals a minimum IFT in the 1 M brine. Please note that these trends may only hold for our particular CRO. CROs across the world vary dramatically in composition; thus, these trends can vary as well.

Although we measure an IFT, this does not mean that the drop interface is an entirely simple one. After a half hour of aging, oscillating pendant drop measurements show a viscoelastic interface with a small Gibbs elastic component (order of 5 mN/m) and a significantly smaller viscous component (order of 0.5 mN/m). The measured viscous component is significantly smaller than the measured Gibbs elastic component, see Supporting Information, Figure S1. Theoretically, both a very slow (time scale ≫ 1/0.06 Hz) exchange of molecules between interface and liquid(s)^43^ and weakly dissipative rearrangements within an irreversibly adsorbed layer^44^ could be responsible for the predominantly elastic behavior. In both scenarios, the oscillations in the drop area are practically equivalent to oscillations in the area per adsorbed species, and we can think of our interface as a quasi-solid layer which slowly accumulates during early aging. Rigorous proof of irreversible adsorption might be possible using setups that allow very careful exchange of the ambient liquid.^45,46^

As aging progresses, the IFT as measured by the Young–Laplace equation does not change significantly; however, the viscoelasticity of the interface increases on the time scale of hours.

### Regions 2 and 3—Liquid Elastic Fits

After a set aging time, we begin drop retraction (i.e., compression of the interface). During early retraction, the IFT decreases and the Young–Laplace fit error remains low. An IFT decrease is expected since we are decreasing the surface area available for surface-active molecules.

The traditional way to visualize the IFT response during drop retraction is through a plot of surface pressure (IFT_0_ – IFT) versus surface area ratio (*A*/*A*_0_).^14^ In Figure 5, we show it as a semilogarithmic plot. Note that the retraction of the drop takes place right to left and that *A* = *A*_0_ corresponds to the aged interface at the start of the compression. The IFT_0_ used to calculate the surface pressure was estimated by taking the first measured IFT of a “freshly injected” drop. Since typically 30 s was needed to generate a drop without causing snap-off, some asphaltene adsorption already occurred within this time, and therefore, the true IFT_0_ will be somewhat higher. The slope of the plot reveals the Gibbs elasticity of the interface (*G* = dγ/d ln *A*, where γ is IFT), under the assumption that the adsorption is irreversible. This representation makes clear that there are, indeed, two distinct regions (regions 2 and 3) with two distinct slopes. In almost every experiment that we performed, these two regions were clearly visible. The only exceptions were conditions where the surface was extremely soft and only showed one slope (i.e., 1 M, 0.5 h aging at high temperature).

This type of surface pressure isotherm plot has been used in previous work on CRO/brine interfaces as evidence for a surface phase transition between two liquid surface phases, a softer liquid expanded phase transitioning to a stiffer liquid compressed phase.^14^ Although we see experimental confirmation of these two regimes, there is some evidence suggesting that a surface pressure isotherm may not be sufficient to describe the surface phases.

The Gibbs elasticity has strict limitations on its use. It assumes that there is no adsorption/desorption at the surface on the time scale of the experiment, and it assumes that the surface shows a liquid shape fittable by Young–Laplace. We feel confident in making the first assumption because we separately performed oscillating pendant drop measurements at moments during retraction and found that the viscous component remained extremely small and the elastic component agreed with the measured Gibbs elasticity. Note also that we are not the first group to make this assumption with regards to CRO systems.^14^

However, the second assumption of a liquid interface may not hold. The DataPhysics OCA fitting software that we used for these isotherms found relatively good Young–Laplace fits in region 3. However, the fitting software used for the elastic shell model could distinguish an increase in the fitting error from region 2 to region 3. These phenomena may be linked because as the Young–Laplace fit gets progressively worse, the changes of surface tension as a function of drop area may also suffer from a loss of significance.

This increase in fit error is the first sign of anisotropic or inhomogeneous surface stresses and thus indicates the onset of a new source for localized surface stress contributions. We will discuss the implications of this in the next section.

### Region 3—Solid Elastic Fits

We quantify the importance of anisotropic and inhomogeneous surface stresses during the deflation of the drops by comparing the residual fit error of a purely liquid elastic fit to that of a solid elastic theory, which includes anisotropic and inhomogeneous surface stress contributions. It is important to emphasize that both the purely liquid elastic and the solid elastic interface theories are mere approximations of more complex interfaces; both theories capture entirely different physical properties and the experimental system might have characteristics correctly described by either of the theories. Thus, we can quantify only which theory has a smaller residual error to hint at the relative importance of the characteristics contained in each theoretical description.

We start our analysis for the solid elastic model by fitting a range of shapes via the purely liquid analysis (see Figure 6a,b) and the circles in (c), where we see that the residual error Figure 6c is fairly low until a critical deformation is reached (*A*/*A*_ref_ = 1 in Figure 6). This is the point we identify as the onset of anisotropic and inhomogeneous surface stress contributions (the boundary between region 2 and region 3) after which the shape of the experimental system might no longer be adequately described by the Young–Laplace equation. The liquid drop shape at this critical deformation can be used as a liquid reference shape for the elastic corrections we apply next since it is the last shape properly characterized by the Young–Laplace fit.

From the purely liquid drop analysis performed for the deformation sequence we also extract the relevant nondimensional properties to fully characterize those liquid shapes. It is evident from Figure 6b that the parameters of the liquid shapes change even *before* a significant increase in fit error is observed. This is consistent with a fluid elasticity, that is, a Gibbs elasticity, until the critical deformation is reached. Once the critical deformation is reached, the experimental shapes differ significantly from any shape accessible through the solution of the Young–Laplace equation and we expect to improve the fit by allowing anisotropic and inhomogeneous tensions.

The increase in Young–Laplace fit error at *A*/*A*_ref_ = 1 coincides with the observed change in the Gibbs modulus between regions 2 and 3. We achieve significantly better fits with the solid elastic theory and a reference shape at the critical deformation, that is, the boundary between region 2 and region 3 (*A*/*A*_ref_ = 1 in Figure 6). This is clearly evidenced by the significantly decreased fit errors in Figure 6c. This suggests that the boundary between regions 2 and 3 indeed marks the onset of non-negligible anisotropic and inhomogeneous surface stress contributions, which are taken into account in solid elastic theory. Note also that the shapes of our pendant capsules are not the same on deflation and reinflation (Supporting Information, Figure S2), which is suggestive of fracturing of a solid layer rather than a surface obeying an EoS model.

Fitting the elastic constitutive model from eq 10 results in fits, which can be seen in Figure 6a. As an example, an additional visualization is included in the Supporting Information. In total, we fit the theory to three different experimental shape sequences at three different temperatures *T* (296, 333, and 358 K), respectively. All values of the elastic control parameters acquired by the fit are shown in Supporting Information, Figure S3; here, we will only focus on the average values over a respective deformation sequence.

### Exploration of the Experimental Parameter Space

The simplicity of retraction experiments means that they can be used to cover a large parameter space of experimental conditions. For this exploration, we choose three temperatures (23 °C/296 K, 60 °C/333 K, and 85 °C/358 K), three aging times (30 min, 4 h, and 17 h), and four brine concentrations (DI water, 0.1 1, and 4 M).

Since we are interested primarily in the trends in elasticity, we will focus on the results from regions 2 and 3 from the liquid elastic analysis (from the π/*A* isotherm) and region 3 from the solid elastic analysis. Although we do believe that the phase transition from liquid to solid interfacial layer occurs between regions 2 and 3, we will still report the π/*A* isotherm fits in region 3 as reasonable approximations of the Gibbs elasticity as this analysis provides a useful comparison to previous literature.

### Brine Composition

**Figure 7:**
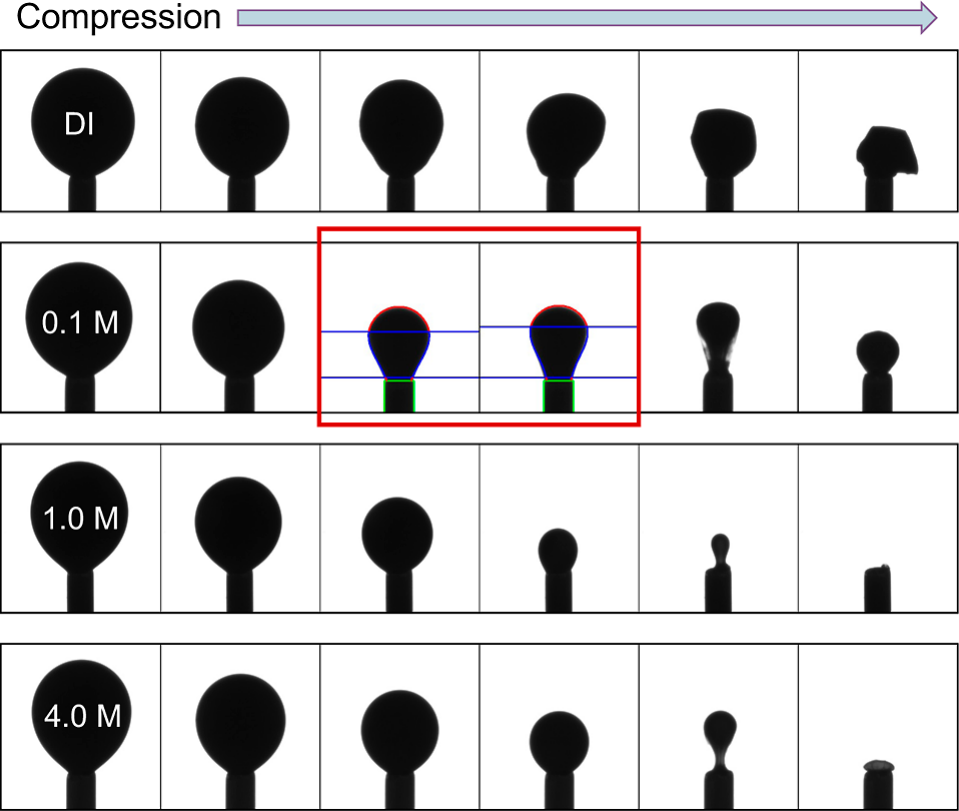
Images for CRO drops compressed at 4 h aging and 85 °C. Trials at different brine concentrations visually show a softening trend. The CRO/DI water interface is so stiff that it crumples completely and asymmetrically. The 1.0 M brine interface is so soft that it barely shows any crumpling at all. The 0.1 M brine is in the “ideal zone” where crumpling is symmetric and can be analyzed. There is a possible minimum in stiffness with 1.0 M brine.

**Figure 8:**
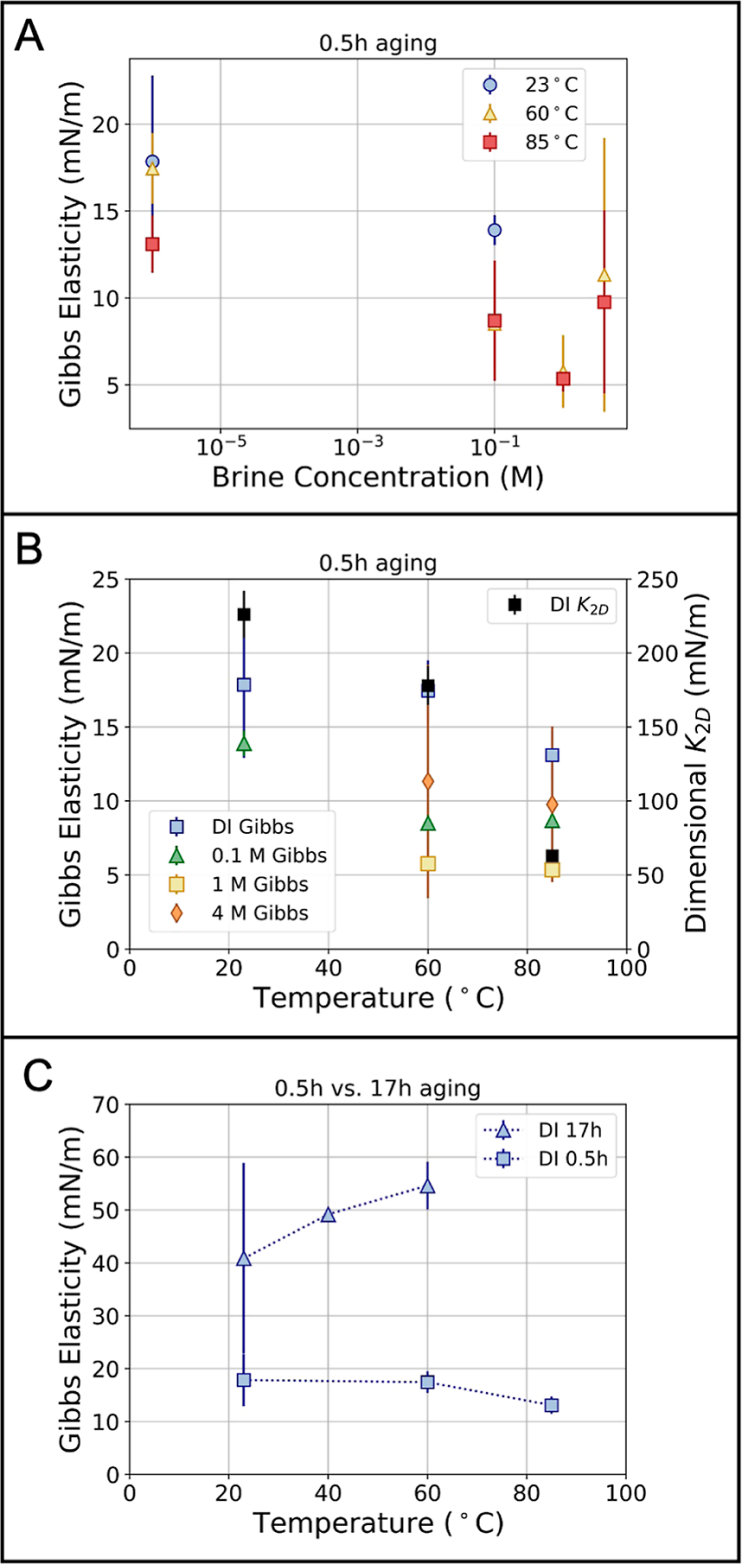
(A) Region 3 Gibbs elasticities vs brine concentration for three different temperatures at 0.5 h aging. The interface shows the same softening at higher brine concentration that was observed visually in Figure 7. (B) The four colors (primary *y*-axis) show region 3 Gibbs elasticities vs temperature for the four brine concentrations at 0.5 h aging. The black points (secondary *y*-axis) show the dimensionalized *K*_2D_ values vs temperature for DI water at 0.5 h aging. All brines show the same qualitative softening trend with increasing temperature; however, the *K*_2D_ values are an order of magnitude higher than the corresponding Gibbs elasticities for DI water. (C) Region 3 Gibbs elasticities vs temperature for DI water at two different ages. After 17 h of aging, the temperature trend is inverted and the interface becomes stiffer with increasing temperature. The interfaces are also generally stiffer after longer aging.

The most dramatic trend we observe is the effect of the brine concentration (Figure 7). At low salinity and high temperature, the CRO/brine interface is very stiff. So stiff that the shapes often suffer from nonaxisymmetric crumpling upon deflation. As the salinity is increased to 0.1 M, the interface softens dramatically, and the capsules can be fitted for the compression modulus in region 3. By the time we reach 1 M, the interface is so soft that wrinkling and crumpling are barely detectable. However, at 4 M, the interface becomes slightly stiffer again. Comparing Figures 7 and 8A, these visual observations are in good agreement with the Gibbs elasticities from region 3 for these different brines.

It is clear from these brine concentration observations that not all experimental conditions are optimal for solid elastic shape fitting. We see such a wide range of stiffnesses that some surfaces are too stiff for the fitting and some are too soft. Surfaces that are too stiff may crumple too asymmetrically for good fits. Surfaces that are too soft will not deviate significantly from the Young–Laplace model. Because the experiments are rather straightforward to perform, we can scan the parameter space for “ideal” conditions where this fitting is accessible to us.

### Temperature

One example of such an ideal condition, where elastic membrane fitting can be used, is CRO aged in DI water at room-temperature.

Analyzing the Gibbs isotherms in region 3, we see a softening of the interface with temperature for drops aged in DI water for 0.5 h (Figure 8B). However, when the same interfaces have been aged overnight, we see the inverse trend with temperature (Figure 8C). This highlights the complex role of surface history in these systems. There could be a number of reasons for this temperature inversion, but we outline one hypothesis. After only a half hour of aging, the interfacial layers that have been grown at different temperatures may be quite similar since they are likely dominated by the initial, rapid adsorption of material. It is possible, then, that the temperature effect that we are seeing is probing the material properties of that layer. In general, most materials soften at higher temperature. Layers that have been growing for 17 h at different temperatures; however, may have grown different structures. If temperature affects not only the material properties of the membrane but also the rate at which material rearranges at the interface or the rate at which new cross-links are formed, then aging for a long time at different temperatures could result in significantly different materials. In this way, even if any given material might soften with temperature, the membrane grown at 85 °C may be a stiffer material than that grown at 23 °C.

While the half-hour experiments fitted with the solid elastic theory show some noise in their respective *dimensionless* compression modulus *K*_2D_/γ_ref_ (see the Supporting Information), we see that the dimensionless compression modulus is fairly constant for the three temperatures. The results for the dimensionless compression moduli are listed in Table 1.

**Table 1:** Results of Elastic Capsule Fitting for Drops Aged in DI Water for 0.5 h at Three Temperatures^a^

temperature	⟨*K*_2D_/γ_ref_⟩	γ_ref_ (mN/m)	⟨*K*_2D_⟩ (mN/m)	⟨ν_2D_⟩
23 °C/296 K	29 ± 2	7.8	226 ± 16	0.74 ± 0.03
60 °C/333 K	27 ± 2	6.6	178 ± 13	0.68 ± 0.02
85 °C/358 K	30 ± 1	2.1	63 ± 2	0.81 ± 0.02

a Here, we show the average non-dimensionalized compression modulus (*K*_2D_/γ_ref_), IFT of the reference state (γ_ref_), average re-dimensionalized compression modulus (*K*_2D_), and Poisson’s ratio (ν_2D_).

For a fully polymeric network interface, we would expect an explicit temperature dependence of *K*_2D_/γ_ref_ ∝ *T*, simply because the entropic spring constant scales linearly with temperature. A hypothesis for the present results is that the dimensionless compression modulus *K*_2D_/γ_ref_ is *not* explicitly dependent on temperature, hinting at a steric interaction as a reason for the large compression moduli. This would support the hypothesis that at a critical area compression *A*/*A*_ref_, contact between steric constituents on the interface is established, which counteracts further contraction.

Our results in region 3 are not compatible with the assumption of an entropic temperature scaling with *K*_2D_/γ_ref_ ∝ *T*, but they are compatible with a temperature-independent dimensionless compression modulus. If we assume the validity of the temperature-independent compression modulus hypothesis, we can average *all* data points for the dimensionless compression moduli together to get a universal nondimensional compression modulus *K*_2D_/γ_ref_ = 30.0 ± 0.7.

It is important to realize that while the dimensionless compression modulus might show no temperature dependence, the same is not true for the dimensional compression modulus
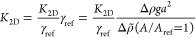
12where we have used eq 5. Thus, the dimensional compression modulus is inversely proportional to , which is different for each temperature trial, as can be seen in Figure 6b.

The IFT of the reference state for the three trials (shown in Table 1) can be used to redimensionalize the dimensionless compression modulus and to give us the dimensional compression moduli (also shown in Table 1). While the dimensional compression modulus is an order of magnitude larger than the fluid elastic Gibbs modulus, it shows the same qualitative, decreasing trend with temperature (plotted together in Figure 8b). The inconsistency of solid elastic compression modulus measurements with liquid elastic Gibbs elasticities is known in literature.^1,36^

In the future, if we could confirm that the dimensionless compression modulus is indeed invariant under temperature changes, we could then construct the dimensional compression modulus without performing additional solid elastic fits by searching for the increase in Young–Laplace fit error and using this critical surface tension as γ_ref_ in eq 12 together with the universal value of the dimensionless compression modulus. This would make future analyses of such surfaces significantly faster and easier. However, we do not yet have the statistics to confirm this invariant compression modulus theory.

Poisson’s ratio seems to also not vary drastically with temperature, as can be seen in the Supporting Information. The average Poisson’s ratios are shown in Table 1. These resulting data for Poisson’s ratio are not compatible with a single constant for the three experiments. If we, regardless of this incompatibility, enforce a constant Poisson’s ratio, we arrive at an overall average ν_2D_ of 0.79 ± 0.01 over all data points.

We plot the apex stresses in the Supporting Information to find that the apex stresses behave similarly for all three temperatures. We can motivate this finding by remembering that the surface stresses are exclusively controlled via the local stretches τ_*s*,ϕ_ = τ_*s*,ϕ_(λ_*s*_, λ_ϕ_); hence, at equal area compression, where d*A* = d*A*_ref_λ_*s*_λ_ϕ_, we expect to find apex stresses similar in magnitude. Thus, the finding of similar apex tension between temperatures is compatible with the claim of temperature-independent dimensionless compression moduli.

It is clear from our analysis of CRO droplets that Gibbs isotherm analysis and elastic shape fitting are not equivalent methods for examining the elasticity in region 3. This highlights the importance of introducing elastic shape fitting for quantitative measurement of the elasticity of the solid layer. These interfaces are extremely complex, and in order to begin to understand their surface structure, we must think critically about the assumptions behind the analysis method we choose to employ. A Gibbs isotherm analysis proves to be very useful to identify the three characteristic regions. It provides correct fits in regions 1 and 2 and can be used to identify the onset of region 3. In the present analysis, elastic shape fitting reduces the error significantly within region 3 suggesting that the CRO interface should be interpreted as a solid rather than a liquid. This insight cannot be gained from a Gibbs isotherm analysis alone. In addition, the temperature dependence of the measured elastic modulus provides hints about the mechanism of solid interface formation and suggests solidification by steric interactions rather than polymeric network formation. We successfully employed a relatively simple elastic constitutive eq 10; for other complex interfaces, more complex constitutive laws might be more appropriate and can, in principle, be also employed in elastic shape fitting.^1^

## Conclusions

In this work, we examine the elastic layers that form at the mutual interface between CRO and aqueous saline solutions. A pendant drop of CRO in such a brine begins as a liquid interface adhering to the Young–Laplace equation. With time, it develops a viscoelastic interfacial layer likely composed of surface-active asphaltenes. Under compression, this viscoelastic layer shows a discontinuous transition from liquid to a solid elastic membrane.

In the viscoelastic fluid regime, we can use standard methods of dilatational rheology to quantitatively measure the viscoelasticity. However, once the layer has transitioned to a solid elastic membrane, such techniques are no longer completely valid. Although such elastic membranes have been reported in the literature, no quantitative measurements have been made of solid layer elasticity. Here, we show that quantitative measurements of the surface elasticity in solid regimes are possible by using shape-fitting elastometry. Not only does this elastometry allow us to measure the elasticity of the compressed layers, but it also gives us clearer insights into where this solid phase transition occurs. This analysis shows that it is likely that the compressed layer becomes solid much earlier in compression than previously assumed.

These compressed layers could play an important role in a number of systems since various types of dynamic flow can cause compression. These systems could show quite different properties of emulsification, adhesion of droplets, capillary pressures, pore flows, and many others. We show that the mechanical properties of these interfaces also have a dependence on brine composition, aging time, and temperature. The stiffest layers were seen at longer aging times and lower salinity, with an ambiguous temperature trend. Further work using this type of quantitative assessment of solid layer regimes is not only fundamentally interesting but could lead to a better understanding of interfacial interactions in oil/water systems.

## Supporting Information Available

CRO composition analysis, brine composition analysis, densities of CRO and brine phases, typical oscillating pendant drop result, capsule shapes during deflation and subsequent reinflation, and fit parameters for Young–Laplace and elastic contributions (PDF)
