# Supplementary material for: Elastometry of Complex Fluid Pendant Capsules
Source: Langmuir. 2023 Nov 8;39(46):16303–14. doi: 10.1021/acs.langmuir.3c01845 (PMC10666541; doi:10.1021/acs.langmuir.3c01845)
Supplement: Supplementary file 1 — la3c01845_si_001.pdf [file la3c01845_si_001.pdf]

Supporting Information:
Elastometry of Complex Fluid Pendant Capsules

Amy Z. Stetten[1],Felix S. Kratz[2], Nathalie Schilderink[1], Subhash Ayirala[3], Michael H.G. Duits[1], Jan Kierfeld[2], Frieder Mugele[1]

[1] University of Twente, Physics of Complex Fluids group, 7500 AE Enschede, The Netherlands
[2] TU Dortmund University, Department of Physics, 44221 Dortmund, Germany
[3] Saudi Aramco, EXPEC Advanced Research Center, Dhahran 34465, Saudi Arabia

Correspondence: m.h.g.duits@utwente.nl

In this document we present the chemical properties of the used crude oil and brines, along with physical properties as were used in the data analysis. Example measurements are shown of small-amplitude oscillatory area deformations of freshly made drops, and of large-amplitude shape and area deformations of drops with an elastic skin. In the last Figure we show the details of fitting the drop shapes to our mechanical model, for 3 temperatures. As part of it, the 2D Poisson ratio and the normalized 2D compression modulus and apex stress are plotted for a range of relative area compressions.

viscosity (at 21.5 °C)	18 mPa s
acid number	0.2 ± 0.04 mg KOH/g
base number	1.1 ± 0.1 mg KOH/g
asphaltenes	2.6 ± 0.3 mass%
C10+	91 ± 3.3 mass%
n-heptane	0.9 ± 0.4 mass%
n-octane	1.0 ± 0.2 mass%
n-hexane	0.6 ± 0.5 mass%

Table S1: Crude oil compositional analysis.

	**Formation Water**	**High Salinity Injection Water**
Na^+ (ppm)	59491	18345
Ca^{2+} (ppm)	19040	650
Mg^{2+} (ppm)	2439	2117
Cl^- (ppm)	132060	35534
SO_4^{2-} (ppm)	350	429
HCO_3^- (ppm)	354	696
Total dissolved solids (ppm)	213734	57872
Ionic strength (M)	4.3	1.1
pH	6.8	7.2

Table S2: Brine composition analysis. Note that our third brine, SmartWater, is simply 10x diluted High Salinity Injection Water.

Temperature	Phase	Density (g/cc)
23 °C	CRO	0.877
	DI Water	0.998
	Smart Water	1.000
	High Salinity Injection Water	1.036
	Formation Water	1.144
60 °C	CRO	0.855
	DI Water	0.983
	Smart Water	0.985
	High Salinity Injection Water	1.021
	Formation Water	1.127
85 °C	CRO	0.833
	DI Water	0.963
	Smart Water	0.970
	High Salinity Injection Water	1.006
	Formation Water	1.110

Table S3: Densities of CRO and brine phases.

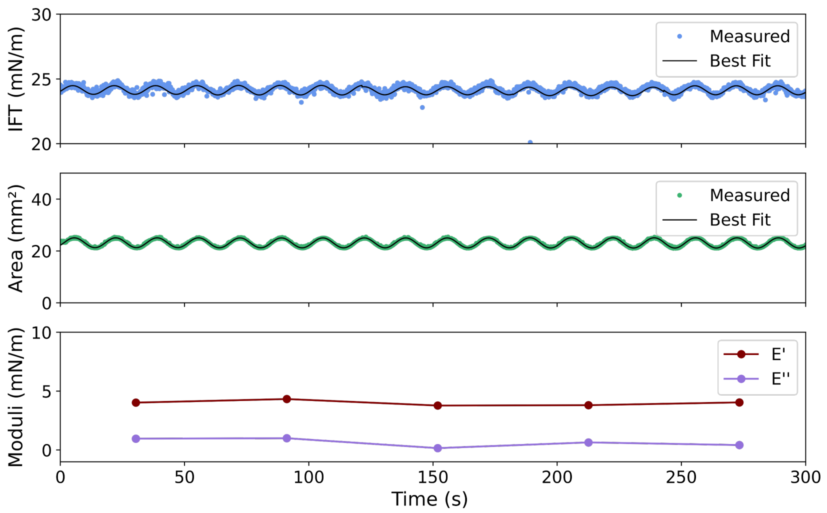

Figure S1: Sample oscillating pendant drop results. Oscillations in drop area and IFT are measured over time. Each successive increment of 600 points (40s at 0.06Hz oscillation) is fit to a sinusoid and elastic and viscous moduli are extracted for a time-running measurement of viscoelasticity.

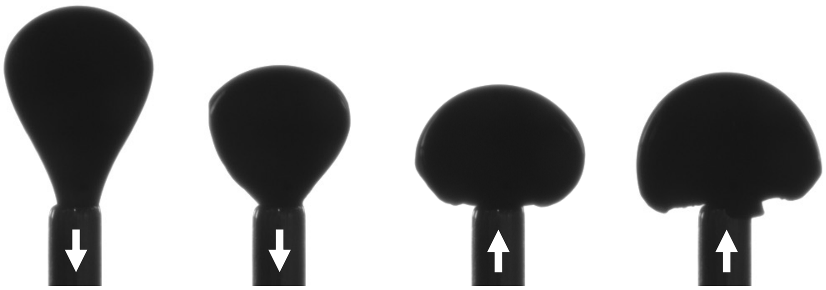

Figure S2: Example of the phenomenon of pendant capsule shape being different on deflation and re-inflation. The first two frames are deflation, the third and fourth are re-inflation. The capsule is clearly different in shape and character.

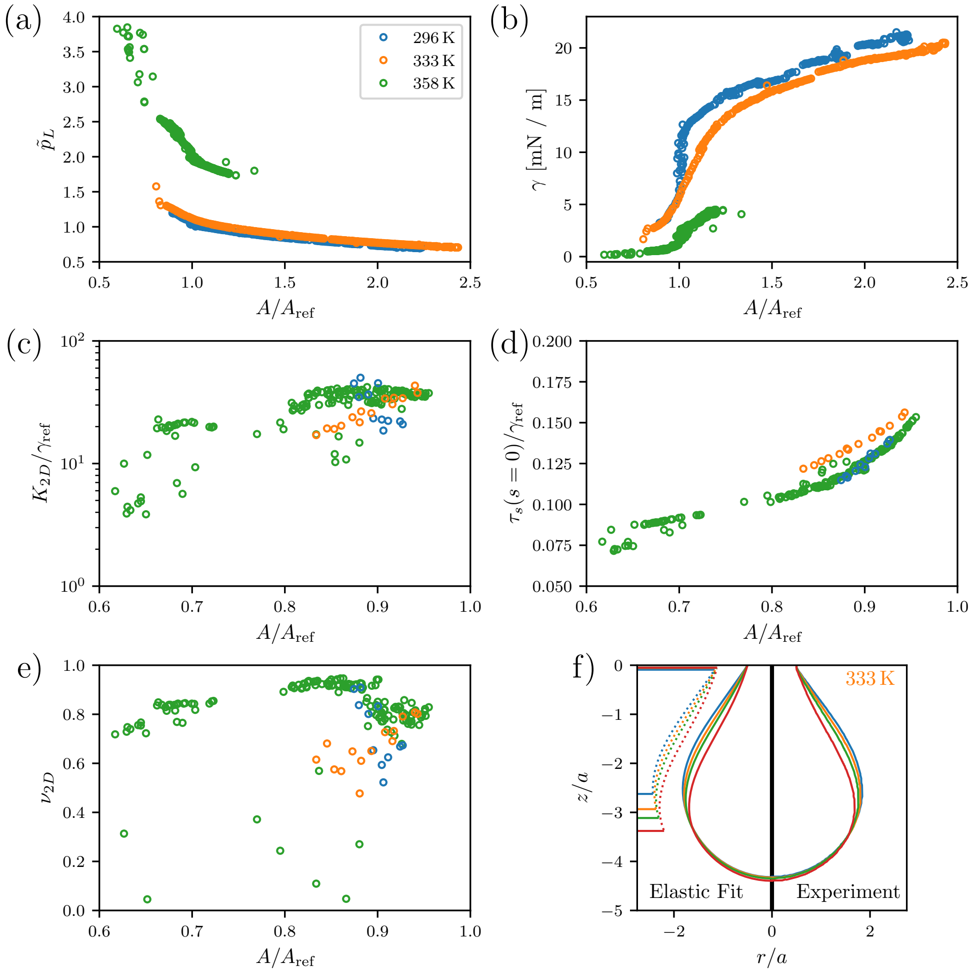

Figure S3: Results for the additional Young-Laplace fit parameter ($\tilde{p}_L$) and the additional elastic fit parameters ($K_{2\text{D}}$, $\nu_{2\text{D}}$ and $\tau_s(s=0)/\gamma_{\text{ref}}$). We plot the relevant control parameters as a function of the relative area compression A/A_{ref}, where A_{ref} is the area of the shape after which the shape error (RMSE) for Young-Laplace fits start to drastically increase, as can be seen in Fig. 6 (c). The non-dimensional control parameter $\tilde{p}_L$ is shown in (a), it is the dimensionless pressure at the apex of the liquid drop. Additonaly, we plot the redimensionalized versions of Fig. 6 (b), by utlilizing eq. (5) together with Table S3 to recover the dimensional surface tension during the deflation (b). The compression moduli for the three trials are shown in (c), Poisson's ratio acquired from the elastic fit is shown in (e) and the dimensionless apex stress in (d). Only the full set of parameters $\{\tilde{p}_L(A/A_{\text{ref}}=1), \Delta\tilde{\rho}(A/A_{\text{ref}}=1), K_{2\text{D}}, \nu_{2\text{D}}, \tau_s(s=0)/\gamma_{\text{ref}}\}$ determines an elastic shape uniquely. We show an additional visualization of the elastic fits in (f).
